# Microgravity-induced transcriptional reprogramming in embryonic chicken limb bud-derived chondrogenic cultures

**DOI:** 10.3389/fcell.2026.1746313

**Published:** 2026-01-21

**Authors:** Patrik Kovács, Zhangzheng Wang, Tibor Hajdú, Krisztián Zoltán Juhász, Éva Katona, Roland Takács, Judit Vágó, Róza Zákány, Szilárd Póliska, Péter Szentesi, László Csernoch, Csaba Matta

**Affiliations:** 1 Department of Anatomy, Histology and Embryology, Faculty of Medicine, University of Debrecen, Debrecen, Hungary; 2 Genomic Medicine and Bioinformatics Core Facility, Department of Biochemistry and Molecular Biology, Faculty of Medicine, University of Debrecen, Debrecen, Hungary; 3 Department of Physiology, Faculty of Medicine, University of Debrecen, Debrecen, Hungary; 4 HUN-REN Cell Physiology Research Group of University of Debrecen, Debrecen, Hungary

**Keywords:** chondrogenesis, mechanical stimuli, microgravity, osteoarthritis, RNA sequencing

## Abstract

**Introduction:**

Extended exposure to microgravity, such as experienced during spaceflight, significantly alters the mechanical environment of skeletal tissues, impacting cartilage development and function. Mechanical unloading disrupts the balance of cellular signaling and extracellular matrix synthesis in cartilage precursor cells, but the molecular consequences and temporal dynamics of these alterations remain incompletely understood.

**Methods:**

We employed simulated microgravity via a random positioning machine (RPM) to investigate stage-specific transcriptomic and phenotypic responses in chondrogenic micromass cultures derived from embryonic chicken (*Gallus gallus*) limb bud cells. RNA sequencing, bioinformatic pathway analysis, and protein interaction network construction were performed on cultures exposed to microgravity for early (days 0–3), late (days 3–6), and continuous (days 0–6) periods.

**Results:**

Continuous microgravity exposure resulted in robust differential expression of 648 genes (adjusted *p*-value <0.05, |log2 fold change| > 1), including suppression of canonical chondrogenic markers (*SOX9, COL2A1*) and upregulation of catabolic enzymes (*MMP13*, ADAMTS family). The affected key signaling pathways included disrupted TGF-β/BMP balance, Wnt/β-catenin activation, and cytoskeletal remodeling. Early and late exposures showed consistent gene expression trends but fewer statistically significant changes. Notably, adrenergic beta receptor 1 (*ADRB1*) was consistently upregulated across all time points.

**Discussion:**

These findings demonstrate that simulated microgravity rapidly induces reversible molecular and cellular adaptations related to cartilage homeostasis and mechanotransduction in this chondrogenic model system. The RPM platform offers a powerful tool to dissect chondrogenesis, cartilage biology, and lineage plasticity under mechanical unloading, providing insights with broad relevance to skeletal tissue mechanobiology.

## 1 Introduction

Humanity faces significant challenges in undertaking extended spaceflight missions lasting months or years, with microgravity posing a major concern. On Earth, appropriate mechanical loading and gravitational forces are fundamental to maintaining the function and structural integrity of skeletal tissues such as bone, cartilage, and muscle (Kohrt et al., 2009). Prolonged exposure to microgravity during space travel leads to substantial musculoskeletal deterioration in astronauts, including muscle atrophy, bone loss, and cartilage dysfunction (Juhl et al., 2021). Although some of these changes may be reversible upon return to Earth and its gravity, extended microgravity-induced alterations can predispose individuals to long-term musculoskeletal disorders, including osteoarthritis (OA) (Hardy, 2024). Consequently, understanding the molecular mechanisms by which microgravity affects skeletal tissues is essential to identify pathways leading to pathological functional and morphological changes.

OA is the most prevalent musculoskeletal disorder worldwide, affecting an estimated 600 million people as of 2021, with projections suggesting a 75%–80% increase in cases by 2050 due to aging populations, rising obesity rates, and lifestyle factors (Collaborators, 2023). OA is characterized by progressive cartilage degradation (Matta et al., 2025), subchondral bone changes, and chronic joint inflammation, leading to pain, functional impairment, and substantial healthcare costs. Despite its enormous socioeconomic burden, OA remains incurable, with no disease-modifying osteoarthritis drug (DMOAD) successfully navigated clinical translation (Li et al., 2023). Alarmingly, despite significant investment and advances in drug discovery, over 95% of phase III DMOAD candidates since 2010 have failed to meet clinical endpoints, often due to insufficient efficacy or off-target effects that were not anticipated during preclinical development (Jiang et al., 2024). This high attrition rate highlights critical deficiencies in current preclinical models that poorly replicate human joint mechanobiology, and a lack of validated, predictive biomarkers for therapeutic monitoring (Reihs et al., 2025).

Given the complex interplay of mechanical loading, inflammation, and cartilage metabolism in OA pathogenesis, there is an urgent demand for mechanistically grounded preclinical platforms that realistically recapitulate the dynamic joint environment. Mechanical loading plays a critical role in cartilage homeostasis and disease (Juhasz et al., 2014; Bader et al., 2011). Loss of physiological loading, as occurs during immobilization or prolonged bed rest, accelerates cartilage degradation and predisposes to OA development (Fitzgerald, 2017). However, current preclinical explant or cytokine-driven models often oversimplify OA’s complexity and do not adequately recapitulate the effects of mechanical unloading on chondrocyte biology or matrix integrity, limiting mechanistic insights into how altered mechanical environments drive OA-like changes (Jeon et al., 2013; Eskelinen et al., 2020).

The random positioning machine (RPM) is a state-of-the-art platform for probing the effects of mechanical unloading. By averaging gravitational vectors through continuously rotating cell cultures along multiple axes, it effectively simulates microgravity conditions to mimic mechanical unloading. Alongside various experimental designs such as rotary bioreactors (Wu et al., 2013) and microgravity facilitated by parabolic flights (Aissiou et al., 2023), RPM exposure has proven useful for studying mechanosensitive pathways in various cell types, including chondrocytes and chondrogenic progenitor cells (Pardo et al., 2005; Wuest et al., 2015). RPM exposure induces rapid, OA-like transcriptomic and phenotypic adaptations in chondrocytes and chondrogenic cultures, marked by cytoskeletal reorganization, catabolic enzyme upregulation (such as MMP-13 or ADAMTS5), and suppressed anabolic signaling (i.e., *SOX9, COL2A1*) (Aleshcheva et al., 2013; Wehland et al., 2020).

Studies using RPMs have illuminated conserved mechanotransduction pathways dysregulated both in OA and mechanical unloading contexts, such as disrupted integrin clustering and Wnt/β-catenin signaling, and PI3K/AKT pathway modulation (Aleshcheva et al., 2013; Abdelfattah et al., 2024; Corydon et al., 2023; Ma et al., 2012). RPM models also capture the concept of “mechanical memory” in chondrocytes: transient unloading phases (3–4 days) provoke reversible phenotypic shifts, whereas prolonged exposure triggers irreversible extracellular matrix (ECM) remodeling, offering a tunable and physiologically relevant *in vitro* system to study OA progression and cartilage repair dynamics (Wehland et al., 2020).

In this study, we employed RPM-exposed chicken chondrogenic micromass cultures to dissect stage-specific transcriptional and phenotypic responses to mechanical unloading, spanning from early differentiation (days 0–3) through matrix maturation phases (days 3–6). Given that developmental programs and key signaling pathways are highly conserved among vertebrates, the embryonic chicken (*Gallusgallus*) micromass system provides a reliable and practical model for studying cartilage development and chondrogenic differentiation *in vitro*. This system has been extensively used to investigate chondrogenesis, ECM formation, and mechanobiological regulation under controlled experimental conditions (Juhasz et al., 2014; Takacs et al., 2023a; Takacs et al., 2023b). We demonstrate that simulated microgravity disrupts the balance of transforming growth factor beta/bone morphogenetic protein (TGF-β/BMP) signaling and induces ECM degradation reminiscent of clinical OA. Notably, these changes were detectable within 72 h, highlighting the utility of simulated microgravity as an expedient, high-fidelity model system for OA mechanobiology and therapeutic development.

## 2 Materials and methods

### 2.1 Experimental setup

The primary objective of this study was to evaluate simulated microgravity by assessing its effects on chondrogenesis and global gene expression in a micromass culture system. Chondrogenic cultures were established from embryonic chicken limb bud mesenchymal progenitor cells (LMPs) and exposed to simulated microgravity (∼0 *g*) using a random positioning machine (RPM). We used distal limb buds from 4.5-day-old chicken (*Gallus gallus*) embryos collected at random from incubated eggs. At this embryonic stage, routine sex determination is not feasible in our workflow; thus, each micromass culture represents a mixed population of male and female cells, assumed to be present in approximately equal proportions.

**FIGURE 1:**
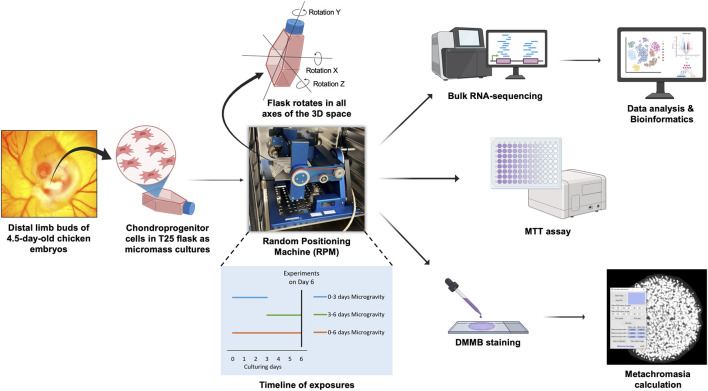
Experimental workflow.

To assess the effects of simulated microgravity at different stages of chondrogenic differentiation, cultures were subjected to RPM during the early phase (days 0–3), the late phase (days 3–6), or throughout the entire 6-day-long culture period (long-exposure group; days 0–6). Cartilage ECM production was evaluated using dimethyl methylene blue (DMMB) staining, cell viability was determined by MTT assay. To investigate transcriptomic changes, bulk RNA sequencing was performed on samples collected on day 6 of culture, and subsequent gene expression and pathway analyses were conducted using R (Figure 1). Data presented are from three biological replicates (N = 3), with one technical replicate to eliminate batch effects. Where applicable, results are expressed as mean ± standard error of the mean (SEM). Pairwise comparisons were evaluated statistically using Student’s t-test.

### 2.2 Chondrogenic micromass cultures and simulated microgravity

Chondrogenic micromass cultures were established from embryonic chicken LMPs using a standardized protocol for spontaneous chondrogenesis (Takacs et al., 2023a). Distal regions of all four limb buds were dissected from early-stage chicken embryos (Hamburger–Hamilton 22–24), pooled, and enzymatically dissociated into a single-cell suspension using trypsin–EDTA for 55 min. Cells were resuspended in Ham’s F12 medium (Gibco, Thermo Fisher Scientific) supplemented with 10% fetal bovine serum (FBS, Gibco) and plated as 100 µL micromass droplets (1.5 × 10^7^ cells/mL) into T25 flasks (9 droplets per flask) equipped with gas-permeable caps to allow gas exchange. Following a 2-h adhesion period in an incubator at 37 °C and 5% CO_2_, cultures were gently flooded with fresh medium to maintain three-dimensional architecture, ensuring complete filling of the flask without bubble formation.

To investigate stage-specific microgravity effects, micromass cultures were exposed to simulated microgravity (∼0 *g*) using an RPM (YURI GmbH) during three intervals: early differentiation phase (days 0–3), late differentiation phase (days 3–6), or continuous exposure (days 0–6). Control cultures maintained identical Ham’s F12-filled flask conditions but remained static in the same incubator without rotation. All experimental groups were terminated at day 6, with early-phase microgravity cultures experiencing standard gravity conditions during days 3–6 before downstream processing.

### 2.3 Sample processing and analysis at day 6

All four groups; i.e., early phase (days 0–3), the late phase (days 3–6), and long-exposure groups (days 0–6), as well as the untreated control cultures were processed on day 6 of culture, when all cultures were terminated. Cartilage ECM production and cell viability were assessed on day 6 using DMMB staining and MTT assay, respectively. Fixed micromass cultures were stained with DMMB (pH 1.8) to quantify metachromatic sulphated glycosaminoglycans, while parallel cultures were subjected to MTT assay to measure mitochondrial activity. In the ECM production assay, DMMB used at low pH selectively binds highly negatively charged sulfated glycosaminoglycans within the ECM of cartilaginous nodules but does not stain nuclear DNA. MTT assay and MATLAB-based metachromatic area quantification (referred to as metachromasia index) followed previously published standardized protocols (Takacs et al., 2023a), with results normalized to static controls.

Total RNA extraction was performed using TRI Reagent (Applied Biosystems) following an established protocol (Takacs et al., 2023c). Chloroform (20% v/v) was added to homogenized samples before centrifugation at 10,000 × *g* for 20 min at 4 °C. RNA was precipitated through incubation at −20 °C in isopropanol for 1 h, followed by resuspension in RNase-free water (Promega) and storage at −80 °C.

Bulk mRNA sequencing followed the previously validated workflow (Takacs et al., 2023b; Takacs et al., 2023c). RNA integrity was verified using a 2100 BioAnalyzer (Agilent Technologies) with the Eukaryotic Total RNA Nano Kit, retaining samples with RNA integrity numbers (RIN) > 8 for library preparation. Sequencing libraries were constructed using the Ultra II RNA Sample Prep Kit (New England BioLabs), followed by single-end 75-cycle sequencing on a NextSeq500 (Illumina) platform. This generated approximately 20 million raw reads per sample, ensuring sufficient depth for differential gene expression and pathway enrichment analysis.

Raw sequencing data (fastq files) were mapped to the *Gallus* reference genome version GRCb7b using HISAT2 aligner and BAM files were generated. Downstream analysis was performed by using StrandNGS (https://www.strand-ngs.com/) software. BAM files were imported and the integrated DESeq algorithm of StrandNGS was used for quantification and normalization. The entire dataset has been deposited and published in the BioProject database (http://www.ncbi.nlm.nih.gov/bioproject/). BioProject ID: PRJNA1282631.

### 2.4 RNA-seq data analysis and bioinformatics

Raw RNA-seq count data were normalized and analyzed for differential expression using the *DESeq2* package (version 1.48.2) in R (version 4.3.1). Genes with low counts were filtered prior to analysis to reduce noise: we only retained genes with raw counts of at least 10 in two or more biological replicates. Counts were normalized using the DESeq2 median-of-ratios method to correct for library size differences. Principal component analysis (PCA) was performed on variance-stabilizing transformation (VST)-normalized data using the *prcomp* function and visualized with *ggplot2* (v4.0.0) to assess sample clustering and replicate consistency.

Differentially expressed genes (DEGs) were identified using the Wald test implemented in DESeq2. Multiple testing correction was performed by calculating adjusted *p*-values (false discovery rate, FDR) using the Benjamini–Hochberg procedure. Genes with an absolute log2 fold change |log2FC| > 1.0 and FDR-adjusted *p*-value <0.05 were considered statistically significant DEGs.

For the primary comparison (continuous microgravity exposure, RPM 0–6 days vs. control), DEGs passing these stringent thresholds (a total of 253 upregulated and 395 downregulated DEGs were identified) were used for all subsequent analyses, including Gene Ontology (GO) and KEGG pathway enrichment via the *clusterProfiler* package (version 4.16.0), and protein-protein interaction (PPI) network construction using the STRING database (version 11.5).

For functional enrichment (GO and KEGG), *Gallus* genes were mapped to their *Homo sapiens* orthologs using the *biomaRt* package (version 2.64.0). Only chicken genes with such human orthologs and that passed expression and filtering criteria were included in the enrichment analysis gene lists and corresponding background universe.

Functional interpretation of DEGs was then carried out with the *clusterProfiler* package, using *H. sapiens* GO and KEGG annotations as the reference, to make advantage of the more comprehensive functional annotation available for human genes. This strategy improves interpretability while maintaining an avian-aware perspective, because enrichment results are interpreted as conserved pathways and processes relevant to vertebrate chondrogenesis and cellular responses to microgravity, rather than as human-specific phenotypes.

In the PPI analysis, the following settings were used: species, *H. sapiens;* minimum interaction confidence score, 0.4. The PPI network was imported into Cytoscape (version 3.10.3) for visualization and hub gene analysis, using degree centrality metrics to identify key regulatory proteins. The top 30 hub genes were selected and visualized to emphasize key regulatory nodes.

The early (RPM on D0–3) and late (RPM on D3–6) exposure comparisons resulted in few or no DEGs passing stringent (adjusted *p*-value <0.05) thresholds. This likely reflects biological variability and reduced microgravity effect within these shorter intervals, as suggested by PCA results indicating less pronounced sample clustering. Therefore, we treat these comparisons as supplementary references, interpreting them with an emphasis on consistent gene expression trends across all groups rather than on individual gene statistical significance alone.

Accordingly, functional enrichment analyses in these short-exposure groups focused on *nominally significant* genes (*p*-value <0.05) to identify potentially relevant biological processes, without attempting high-confidence regulatory network construction or hub gene identification. This stratified approach balances rigor in the main dataset with exploratory insights from shorter treatments. Subsequently, Venn diagrams were employed to identify overlapping DEGs across three comparisons (Bardou et al., 2014). Conserved-directionality DEGs were further isolated through directional Venn analyses and visualized using the *pheatmap* (version 1.0.13) package.

## 3 Results

### 3.1 Phase-dependent modulation of chondrogenesis by simulated microgravity

**FIGURE 2:**
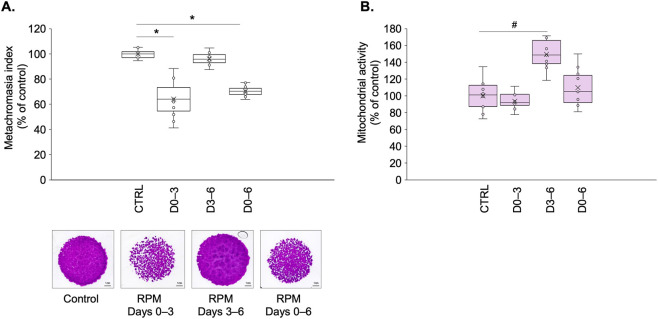
RPM-induced simulated microgravity effects on chondrogenic cultures on day 6. **(A)** DMMB-stained cartilage matrix production following RPM exposure at the following differentiation stages: early (D0–3), late (D3–6), and throughout differentiation (D0–6). Original magnification: ×4; scale bar: 1 mm. Bar graphs show MATLAB-quantified metachromatic areas (metachromasia index; mean ± SEM vs. control). Asterisks (*) indicate *p* < 0.001 (N = 3 biological replicates). **(B)** Cell viability determined by MTT assay (on day 6). Data shown as mean ± SEM (N = 3). Hash symbol (#) denotes *p* < 0.001 vs. control. Statistical significance was assessed using unpaired two-tailed *t*-test.

To evaluate stage-specific effects of simulated microgravity (mechanical unloading), we analyzed metachromatic cartilage ECM production and cell viability on culture day 6 following exposure to microgravity. Quantitative DMMB staining revealed significant reductions in metachromatic sGAG content when cultures experienced simulated microgravity during early chondrogenesis (D0–3: 64% ± 14.9% of control; p < 0.001) or throughout differentiation (D0–6: 70% ± 4.2% of control; p < 0.001) (Figure 2A). Late-stage exposure (D3–6) showed no significant alteration in ECM production (97% ± 5.03% of control; *p* = 0.156), suggesting mechanical signals primarily impact matrix synthesis during initial differentiation phases.

MTT assay results on day 6 also demonstrated temporal specificity in cellular responses. While early and continuous microgravity groups maintained control-level mitochondrial activity, late-exposure (D3–6) cultures exhibited a pronounced (149% ± 17.5%; *p* = 0.008) increase of this parameter versus controls (Figure 2B). This bifurcated response indicates distinct mechanical regulation of ECM production and survival mechanisms during chondrogenic progression.

### 3.2 Global transcriptomic changes induced by stage-specific microgravity exposure

**FIGURE 3:**
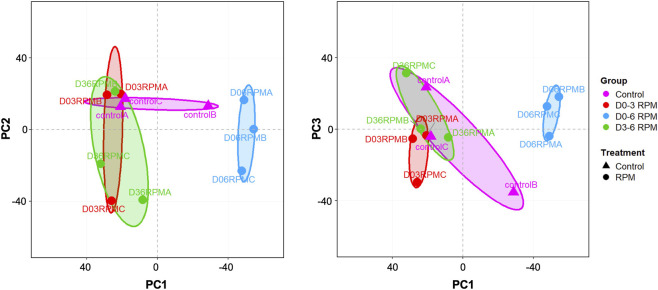
Principal component analysis (PCA) of the bulk RNA-seq data from chondrogenic cultures exposed to simulated microgravity. PCA plots show normalized RNA-seq data distribution across experimental groups (N = 3 replicates/group). Principal component 1 (PC1) separates continuous microgravity exposure from day 0 through 6 (D0–6_RPM) from other conditions. Variance explained: PC1, 38.6%; PC2, 19.5%; PC3, 13.8%. Color coding denotes microgravity exposure windows.

To elucidate the mechanisms underlying microgravity effects on chondrogenesis, we analyzed bulk RNA-seq data through PCA. Figure 3 illustrates the variance structure between experimental groups using normalized expression data, where the first principal component (PC1; accounting for 38.6% of the variance) revealed distinct separation between cultures exposed to continuous microgravity (D0–6) and other experimental conditions. While moderate inter-replicate variability was observed within groups (N = 3), the PCA demonstrated that prolonged microgravity exposure induced substantial transcriptomic reorganization compared to stage-specific interventions or control cultures. PC2 (19.5% variance) and PC3 (13.8% of variance) showed partial separation of late-phase microgravity-exposed cultures (D3–6) from controls and early-exposure groups, suggesting temporally distinct regulatory mechanisms. These findings suggest that simulated microgravity induces significant genome-wide expression changes in chondrogenic cultures, with exposure duration critically influencing transcriptional responses.

### 3.3 Stage-specific transcriptional reprogramming under microgravity

**FIGURE 4:**
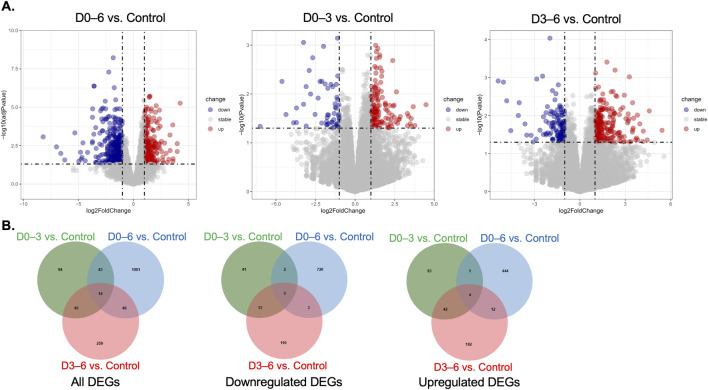
Differential gene expression in chondrogenic micromass cultures following exposure to simulated microgravity. **(A)** Volcano plots depicting differentially expressed genes (DEGs) in each microgravity group compared to control. Each point represents a single gene, with the x-axis representing the log2 fold change and the y-axis representing the statistical significance (–log10 *p*-value). Blue points indicate significantly downregulated genes, red points indicate significantly upregulated genes, and grey points represent genes without marked significant expression changes. **(B)** Venn diagrams illustrating the overlap and distribution of total, upregulated, and downregulated DEGs among the three microgravity exposure groups (N = 3 replicates/group). For consistency, identical criteria were used for directional Venn analyses (P < 0.05; D0–3: 196 DEGs; D3–6: 366 DEGs; D0–6: 1186 DEGs).

RNA sequencing revealed that the most extensive and statistically robust transcriptomic changes occurred in chondrogenic cultures subjected to continuous simulated microgravity exposure throughout the entire differentiation period (D0–6). By applying stringent criteria; i.e., absolute log2 fold change (|log2FC|) > 1 and FDR-adjusted *p*-value <0.05, we identified 648 DEGs in the D0–6 comparison, comprising 253 upregulated and 395 downregulated genes (Figure 4A). These DEGs served as the primary dataset for downstream functional enrichment and protein-protein interaction analyses, permitting high-confidence biological interpretations.

In contrast, the early (D0–3) and late (D3–6) exposure groups yielded fewer DEGs. In fact, no DEGs from these shorter exposures met the stringent FDR-adjusted *p*-value <0.05 cutoff at the same fold change level. This observation likely reflects less pronounced transcriptional responses or greater sample variability within these windows, as suggested by principal component analysis indicating weaker clustering by treatment at these early or intermediate time points. Nevertheless, these comparisons provide a valuable supplemental context.

Rather than focusing solely on statistically significant individual genes in the D0–3 and D3–6 groups, we emphasized consistent gene expression trends observed across all exposure durations, prioritizing the directionality of change (up- or downregulation) to gain insight into early mechanotransductive adaptations. Functional enrichment analyses in these groups were therefore based on nominal *p*-value thresholds (*p* < 0.05, D0–3: 196 DEGs; D3–6: 366 DEGs; D0–6: 1,186 DEGs), focusing on biological processes and pathways showing consistent modulation rather than definitive gene-level regulation. This strategy allowed us to extract meaningful biological insights despite the limited number of DEGs meeting stringent adjusted *p*-value criteria.

**FIGURE 5:**
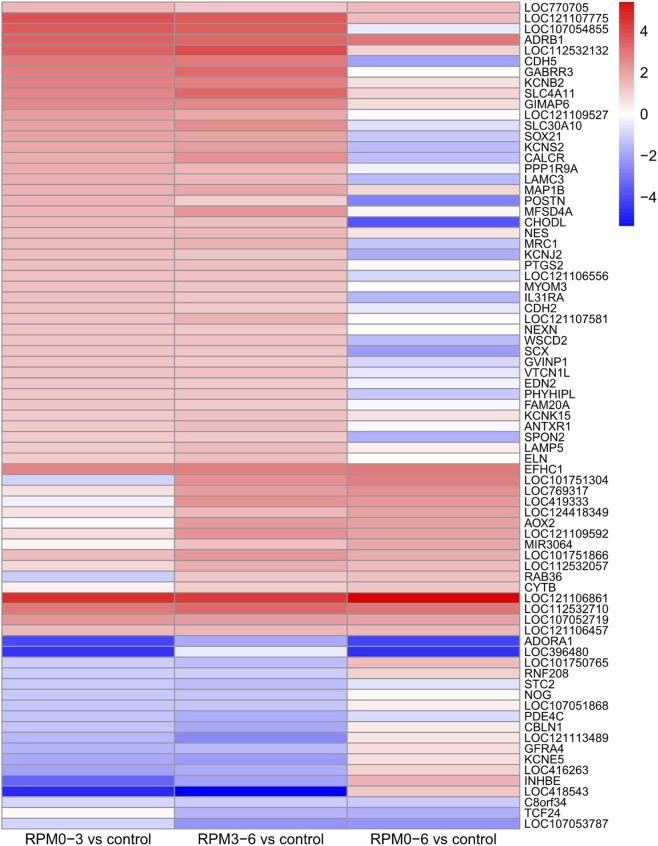
Conserved directional regulation of microgravity-responsive genes across exposure phases. Heatmap visualization of differentially expressed genes (DEGs) showing consistent regulation patterns (*p* < 0.05) in at least two microgravity exposure groups. Rows represent individual genes, columns indicate experimental comparisons (D0–3, D3–6, D0–6 *versus* control). Color intensity corresponds to log2 fold change values, with red indicating upregulation and blue denoting downregulation relative to control. Genes were clustered using unsupervised hierarchical clustering (Euclidean distance, complete linkage) based on their expression profiles across conditions. Only genes maintaining consistent regulation directionality in at least two shared comparisons are displayed. Color scale normalized to maximum absolute fold change across all groups (N = 3 replicates/group).

To elucidate shared transcriptional responses to microgravity, we performed Venn analyses on all DEGs detected in the three comparisons (no adjusted *p*-value cutoff). Among 1,584 total DEGs, only 14 genes were significantly dysregulated across all three exposure windows, with 150 genes intersecting at least two groups (Figure 4B). Complete DEG lists in all three exposure groups are provided in Supplementary Material 1. Subsequent filtering for consistent fold change directionality further refined these to 77 genes that displayed concordant up- or downregulation across multiple groups (Figure 5). This gene set represents core mechanosensitive transcripts responsive to microgravity regardless of exposure duration.

### 3.4 Gene Ontology analysis identifies biological processes affected by simulated microgravity

**FIGURE 6:**
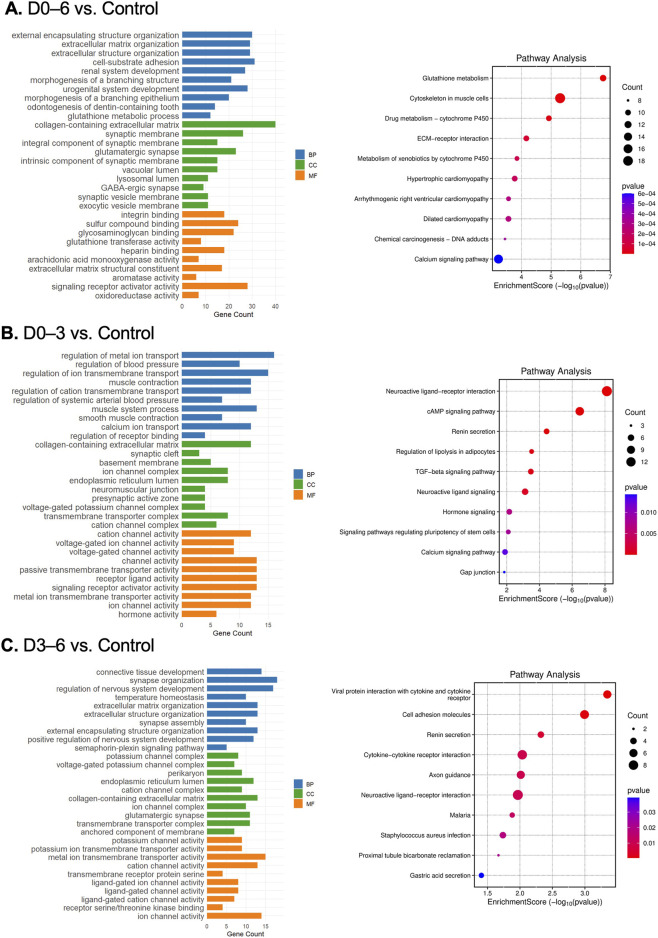
Gene ontology (GO) enrichment and pathway analysis of differentially expressed genes (DEGs) in chondrogenic micromass cultures following simulated microgravity exposure. **(A–C)** GO classifications and KEGG pathway analysis of DEGs (fold change >2, *p* < 0.05) identified in each microgravity exposure group compared to control: **(A)** continuous exposure (D0–6), **(B)** early-stage exposure (D0–3), and **(C)** late-stage exposure (D3–6). For each group, the top 10 terms from the three GO categories: biological process (BP), cellular component (CC), and molecular function (MF) are displayed, ranked by enrichment score. Pathway analysis results are similarly ranked by enrichment score along the *x*-axis. Dot size corresponds to the number of DEGs associated with each term or pathway, while color intensity reflects statistical significance, with warmer colors indicating lower *p*-values. Only terms and pathways meeting the specified significance thresholds are shown (N = 3 replicates/group).

To elucidate the biological processes and molecular functions altered by simulated microgravity, we performed GO enrichment and KEGG pathway analyses on DEGs from each exposure group. For the continuous microgravity exposure group (D0–6), where a substantial number of DEGs were identified (|log2 fold change| > 1 and adjusted *p*-value <0.05), enriched GO terms highlighted key processes relevant to cartilage biology. Notably, ECM organization, morphogenic processes, and growth factor activity-related terms were prominently enriched (Figure 6A). Corresponding pathway analysis revealed significant enrichment of ECM–receptor interaction, calcium signaling, and cytoskeleton in muscle cells, underscoring profound ECM remodeling and mechanotransduction disruptions under prolonged unloading.

In contrast, early-stage (D0–3) and late-stage (D3–6) exposure groups yielded few or no DEGs meeting adjusted *p*-value <0.05 thresholds. Despite this, we performed GO and pathway enrichment analyses on genes passing nominal *p*-value cutoffs, interpreting these results cautiously and focusing on consistent gene expression trends across groups rather than on strict statistical significance of individual genes. For the early-stage group (D0–3), enriched GO terms centered on ECM homeostasis, particularly collagen-containing ECM, as well as ion channel activity (Figure 6B). The late-stage group (D3–6) showed enrichment in connective tissue development, ECM organization, and ion channel activity dominated by potassium channels (Figure 6C). Additionally, pathways related to cell adhesion and cytokine signaling were significantly enriched.

### 3.5 Protein interaction network reveals microgravity-induced pathway dysregulation in chondrogenesis

**FIGURE 7:**
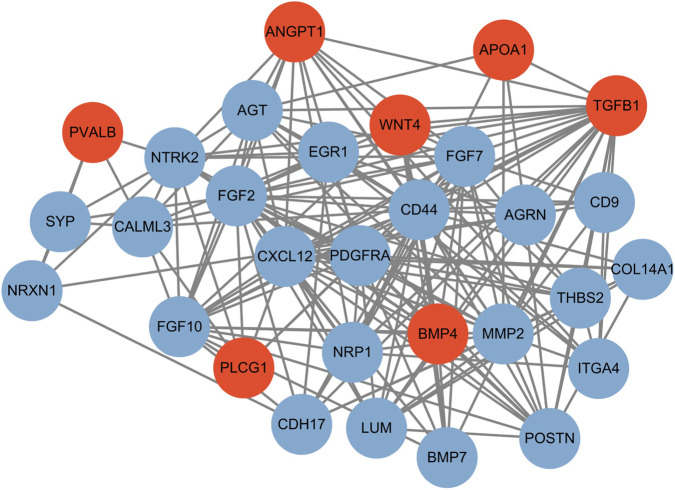
Protein-protein interaction (PPI) network of microgravity-responsive genes in continuously exposed chondrogenic cultures. The network was constructed from the top 30 hub genes selected based on degree values. Nodes represent differentially expressed proteins (red: upregulated; blue: downregulated) with edges indicating functional associations (STRING database v12.0, medium-confidence interactions >0.4). The layout was optimized using force-directed algorithms to emphasize modular organization (N = 3 replicates/group).

To identify drivers underpinning the observed transcriptional alterations, a PPI network was constructed from DEGs that satisfied stringent filtering criteria (adjusted *p*-value <0.05, |log2FC| > 1) in the continuous simulated microgravity group (D0–6). This group demonstrated distinct clustering in PCA analysis and pronounced changes in cartilage ECM synthesis (Figure 7), justifying focused network-level analysis of chondrogenic responses to unloading.

The resulting interactome converged on several mechanistically linked pathways critical to chondrocyte differentiation and function. Growth factor signaling formed a prominent hub, with upregulated TGFB1 and BMP4 alongside downregulated BMP7 and multiple fibroblast growth factors (FGF2, FGF7, FGF10), indicating microgravity-induced reshaping of TGFβ/BMP and FGF signaling landscapes. This pattern suggests altered morphogen gradients that coordinate chondroprogenitor condensation and matrix assembly, with FGF suppression potentially dampening early matrix production and proliferation.

A second major cluster comprised mechanosensing and cell-matrix interaction components, including CD44, platelet-derived growth factor receptor alpha (PDGFRA), integrin alpha-4 (ITGA4), CD9, and adhesion molecules such as cadherin-17 (CDH17) and neuropilin-1 (NRP1). Altered expression of these receptors and co-receptors points to disturbed compromised transmission and signaling cross-talk under unloading, consistent with a reduced capacity of chondrogenic cells to translate mechanical input into biochemical responses.

Matrix-related and matricellular proteins constituted another key module, with downregulation of structural and regulatory ECM components such as COL14A1, lumican (LUM), periostin (POSTN), and thrombospondin-2 (THBS2), agrin (AGRN), and the matrix-remodeling enzyme MMP2, contrasted by upregulation of apolipoprotein A-I (APOA1). Together with changes in angiogenic and vasoactive factors (e.g., angiopoietin-1, ANGPT1 and angiotensinogen, AGT) and WNT4/EGR1 signaling, this pattern is indicative of impaired ECM assembly, heightened matrix turnover, and broader microenvironmental dysregulation under simulated microgravity.

Collectively, this PPI signature under simulated microgravity corroborates the concept of “mechanical unloading syndrome” in chondrogenic progenitor cells, characterized by compensatory upregulation of select growth factors (TGFB1, BMP4), amid widespread downregulation of FGF and ECM-related genes, and a shift toward catabolic remodeling via MMP2. These network-level alterations align with disturbed ECM homeostasis and mechanosensitive signaling, providing a systems view of how sustained unloading perturbs chondrogenic pathways.

### 3.6 Expression profiles of chondrocyte marker genes and OA biomarkers

To evaluate the impact of simulated microgravity on critical chondrocyte-specific genes and established OA biomarkers, we analyzed the expression patterns of key genes in chondrifying cultures across all microgravity exposure groups. The gene set was curated by merging entries from relevant GO categories related to cartilage homeostasis, development, morphogenesis, condensation, and articular cartilage formation, following the approach detailed in our previous study (Takacs et al., 2023b) (see Supplementary Material 1).

Canonical hyaline chondrocyte markers, including *ACAN, COL2A1, COL9A1, COL27A1, COMP, MATN1, MATN3,* and the SOX transcription factor trio (*SOX5, SOX6, SOX9*), displayed distinct temporal expression patterns (Table 1). Both short-exposure groups (D0–3 and D3–6) exhibited general downregulation of these markers, consistent with transient suppression of chondrogenic identity under brief microgravity conditions. In contrast, in the long-exposure group (D0–6), these markers displayed a trend toward upregulation or attenuated downregulation, suggesting an adaptive transcriptional response to sustained mechanical unloading.

**TABLE 1:** Differential expression of selected chondrogenic marker genes, signaling components, and extracellular matrix remodeling factors in response to simulated microgravity. Log2 fold change (log2FC) values are shown for early (D0–3), late (D3–6), and continuous (D0–6) exposure groups relative to static (1 *g*) controls. Positive and negative values indicate upregulation and downregulation under microgravity, respectively.

Symbol	RPM D0–3 log2fc	RPM D3–6 log2fc	RPM D0–6 log2fc
*ACAN*	−0.696	−1.043	0.849
*ADAMTS12*	0.827	0.645	−0.646
*ADAMTS7*	0.069	0.179	0.297
*ADRB1*	3.309	3.258	2.851
*BMP2*	0.166	0.126	0.044
*BMP4*	0.100	0.313	1.464
*BMP5*	0.589	0.375	−2.294
*BMP6*	−0.205	−0.886	0.328
*BMP7*	−0.466	−0.361	−1.541
*BMPR1A*	0.025	0.157	−0.848
*BMPR1B*	−0.490	−0.754	0.415
*BMPR2*	0.318	0.115	−0.308
*COL27A1*	−0.316	−0.472	0.750
*COL2A1*	−0.574	−0.511	1.196
*COL9A1*	−0.629	−0.861	0.801
*COMP*	−0.444	−0.844	0.432
*CTNNB1*	−0.227	−0.110	0.220
*GDF11*	−0.493	0.222	0.447
*GDF15*	−0.344	0.165	0.902
*GDF2*	0.856	−0.004	−0.760
*GDF3*	−0.546	−0.607	0.372
*GDF5*	−0.135	−0.069	−0.814
*GDF6*	0.451	0.638	−1.316
*GDF7*	−0.242	−0.164	−0.584
*GDF9*	0.006	−0.326	−0.630
*MATN1*	−0.368	−0.604	1.596
*MATN3*	−0.507	−0.703	0.744
*MMP13*	0.161	0.841	−0.613
*MMP2*	0.539	0.350	−1.379
*MMP23A*	0.324	0.304	−0.587
*MMP24*	0.255	0.315	−0.080
*MMP27*	−0.162	0.925	1.033
*MMP28*	0.585	1.286	−0.187
*SMAD1*	−0.238	−0.481	−0.438
*SMAD3*	0.128	0.198	0.042
*SMAD5*	0.195	0.152	−0.715
*SMAD6*	0.360	0.178	0.003
*SMAD7*	0.359	0.021	−0.617
*SMAD7B*	0.124	0.253	−0.395
*SMAD9*	0.548	0.231	−0.770
*SOX5*	−0.037	−0.131	0.109
*SOX6*	−0.070	−0.327	−0.148
*SOX9*	0.028	−0.228	−0.193
*TGFB1*	−0.391	0.019	1.041
*TGFB2*	0.378	0.420	−0.392
*TGFBR1*	0.239	0.079	−0.626
*TGFBR2*	0.133	0.103	−0.510
*WNT5A*	−0.040	0.185	0.018
*WNT7A*	0.163	0.753	−2.385
*WNT7B*	0.711	0.974	1.163
*WNT9A*	1.031	1.390	−0.173

Analysis of the BMP signaling pathway revealed nuanced regulatory dynamics (Table 1). *BMP2* and *BMP4* were modestly but consistently upregulated across all groups, while *BMP7* was downregulated under all conditions. *BMP5* and *BMP6* displayed inconsistent changes, with *BMP5* significantly downregulated in the long-exposure group. The BMP receptor *BMPR1A* was uniformly downregulated across all microgravity exposure durations. Notably, SMAD family genes, key downstream mediators of BMP signaling, were upregulated in short-exposure groups but downregulated in the continuous microgravity exposure group, diverging from the classical chondrogenic marker trends.

TGF-β ligands *TGFB1* and *TGFB2* exhibited reciprocal expression patterns: *TGFB1* was downregulated or unchanged in short exposure groups and upregulated in long exposure, while *TGFB2* showed the opposite trend. This was paralleled by downregulation of the TGF-β receptors *TGFBR1* and *TGFBR2* in the long-exposure group. Regarding growth and differentiation factors (GDFs), *GDF5* was consistently downregulated, while *GDF6* mirrored SMAD expression patterns, being downregulated exclusively in the prolonged exposure group (Table 1). These results align with the established roles of GDFs in chondrogenesis, mechanotransduction, and OA pathogenesis (Clarke et al., 2014; Sun et al., 2021).

Within the Wnt signaling pathway, *WNT7B* was upregulated in all groups, whereas *WNT7A* and *WNT9A* were downregulated exclusively in the long-exposure group. Beta-catenin (*CTNNB1*) expression increased with continuous microgravity but decreased under short exposures (Table 1), indicating dynamic regulation of canonical Wnt signaling in response to microgravity exposure duration.

Genes associated with ECM remodeling also demonstrated exposure duration-dependent regulation. *ADAMTS7* and *ADAMTS12* were generally upregulated in response to microgravity exposure, except *ADAMTS12*, which was downregulated in the prolonged exposure group. Several matrix metalloproteinases (*MMP2, MMP13, MMP23A, MMP24*, and *MMP28*) were downregulated only in the long-exposure condition, whereas *MMP27* was consistently upregulated across all exposure groups (Table 1). These patterns suggest a complex modulation of ECM turnover under altered mechanical environments.

Remarkably, the adrenergic beta receptor 1 (*ADRB1*) was consistently and significantly upregulated across all microgravity exposure groups, making it a potential robust biomarker of mechanical unloading in chondrogenic cultures.

## 4 Discussion

This study demonstrates that simulated microgravity, applied *via* random positioning machine (RPM), induces rapid and robust transcriptional and phenotypic changes in chondrogenic micromass cultures. Our data show that mechanical unloading alters key pathways of chondrocyte differentiation and ECM remodeling within just days of exposure, providing a responsive platform for dissecting cartilage mechanobiology and fate decisions.

Our findings reveal a nuanced temporal response of chondrogenic cultures to the timing and duration of simulated microgravity. Both early (D0–3) and continuous (D0–6) exposure resulted in marked suppression of proteoglycan synthesis without compromising cell viability, suggesting that mechanical unloading rapidly impairs anabolic activity prior to affecting metabolic health. In contrast, the late exposure (D3–6) group exhibited a distinct profile with less pronounced suppression, reflecting potential initiation of compensatory mechanisms. This interpretation is supported by our bulk RNA-seq data, where only the continuous exposure group formed a distinct transcriptomic cluster indicative of sustained and coordinated gene expression changes. The early and late time points likely represent transient or intermediate cellular states with more heterogeneous responses. Mechanistically, early unloading suppresses key anabolic regulators, including SOX9 and TGF-β/BMP signaling, promoting catabolic gene activation, while prolonged unloading consolidates these effects into a stable transcriptional program. Late exposure may engage adaptive signaling pathways such as Wnt/β-catenin and cytoskeletal remodeling, facilitating partial restoration of ECM synthesis and cellular function. These temporal dynamics highlight the complexity of chondrocyte mechanotransduction and underscore the importance of exposure duration in shaping cartilage responses to mechanical stimuli.

In line with the above observations, a major finding from our transcriptomic analysis is the stage-dependent reprogramming observed under continuous microgravity: canonical chondrocyte markers (such as *SOX9, COL2A1, ACAN*) and anabolic regulators were significantly suppressed, while genes associated with matrix degradation (e.g., MMPs, ADAMTS family members) and mechanotransduction were upregulated. These findings are consistent with prior work, which highlighted substantial downregulation of SOX trio members (SOX5/6/9), suppression of chondrogenic marker genes, and rapid induction of catabolic enzymes in RPM-exposed chondrocytes and MSCs (Wehland et al., 2020; Duke et al., 1995). Both studies, including ours, identify a compressed timeline for the loss of cartilage identity and the onset of a degradative phenotype, with transcriptomic shifts emerging after 48–72 h of unloading.

These changes mirror clinical disuse scenarios such as prolonged bed rest or post-injury immobilization, where rapid cartilage loss and matrix degradation occur (Drei et al., 2025), validating our model’s translational relevance. The observed shifts identify actionable therapeutic targets including suppression of IL-6/MMP-13 and reinforcement of the SOX/TGF-β axis: targets aligning with DMOAD mechanisms in clinical development (Coricor and Serra, 2016; Hu and Ecker, 2021; Oo et al., 2021).

Our work clearly highlights the tunable nature of the RPM approach: while short-term exposures result in partial and transient suppression, longer exposures evoke more pronounced and sustained changes. Notably, we observe distinct regulatory dynamics among core signaling pathways, such as TGF-β/BMP and Wnt/β-catenin, consistent with reports that mechanical context influences growth factor responsiveness and lineage commitment in chondrogenic and osteogenic models (Wehland et al., 2020). Emerging evidence from simulated microgravity research indicates that unloading-induced lineage shifts in chondrogenic cultures are reversible with subsequent re-loading, reflecting the plasticity of chondrocyte fate under dynamic mechanical environments (Visigalli et al., 2010; Basso and Heersche, 2006).

A unique aspect of simulated microgravity models is the “mechanical memory” of chondrocytes and progenitor populations. Previous RPM studies demonstrate that acute exposure to simulated microgravity rapidly disrupts the chondrocyte phenotype, suppressing anabolic markers (*SOX9, COL2A1*) while upregulating catabolic enzymes (*MMP13*) and inflammatory mediators (*IL6*) within 1–4 days (Wehland et al., 2020; Duke et al., 1995). OA chondrocytes exhibit heightened catabolic responses to unloading, indicating increased susceptibility in OA microenvironments (Lv et al., 2019). Microgravity also impairs osteocyte cytoskeletal architecture and mechanotransduction, altering integrin and Wnt/β-catenin signaling, which are key pathways in OA (Yang et al., 2015). However, reloading or restoration of normal gravity leads to a partial recovery of expression for stemness and chondrogenic markers. Certain differentiation and matrix remodeling changes appear persistent, as earlier studies, in line with our own findings, report upregulation of osteochondral lineage markers and ECM remodeling transcripts after unloading and reloading cycles (Wehland et al., 2020). These results support a model in which cells subjected to mechanical unloading not only lose homeostatic cartilage markers but also gain a propensity for hypertrophic or fibrocartilaginous transitions, aligning with mesodermal lineage flexibility reported under microgravity.

The upregulated catabolic genes and the suppressed anabolic markers parallel the cartilage degradation observed in disuse scenarios, such as immobilization, which balneotherapy aims to counteract through hydrostatic unloading and thermal modulation. Balneotherapy may mimic the mechanotransductive “reloading” phase post-microgravity exposure, where restoring physiological loading reactivates chondrogenic pathways (TGFβ, SOX9) and suppresses inflammation (Green and Scott, 2017). By modeling the transition between unloading (microgravity) and reloading (post-therapy), these systems can identify molecular targets that align with balneotherapy’s documented anti-inflammatory and chondroprotective effects (Cheleschi et al., 2020). This approach bridges *in vitro* mechanobiology with clinical strategies, offering insights into how mechanical modulation, which is central to balneotherapy, can optimize cartilage repair and OA management.

Our data further reveal robust and consistent upregulation of *ADRB1* following microgravity exposure, implicating its role as an early molecular sensor for mechanical state transitions. Given that β1-adrenergic signaling exerts predominant anabolic effects in response to mechanical stimulation (Pierroz et al., 2012), *ADRB1* may serve as a useful biomarker for the mechanical loading status of chondrogenic cultures.

By identifying phase-specific molecular markers and mechanotransduction pathways, our study provides a toolkit for fine-tuning the “dose and timing” of mechanical interventions in preclinical models, enabling precise identification of combination therapy windows by monitoring phase-specific molecular responses.

### 4.1 Limitations of the study

While our study provides valuable insights into the mechanobiological responses of chondrogenic progenitors to simulated microgravity, several limitations warrant consideration. First, we utilized embryonic chick limb bud progenitor cells as a model system. Although this approach offers controlled and reproducible *in vitro* conditions for studying early cartilage formation and mechanosensitive pathways (Takacs et al., 2023a; Takacs et al., 2023b), chick embryonic cells differ from mature human articular chondrocytes, the clinically most relevant cell type for OA. Differences in species, developmental stage, cartilage zonation, and matrix composition may limit direct extrapolation of findings to adult human joint biology (Schulze-Tanzil et al., 2009). Additionally, functional interpretation of transcriptomic changes relied on mapping chicken genes to their human orthologs and using human-centric annotation resources, which, while enhancing interpretability and translational relevance, may introduce bias and should be viewed as reflecting conserved vertebrate pathways rather than human-specific phenotypes. Nevertheless, embryonic avian models remain powerful for fundamental developmental and mechanobiological research due to their accessibility and well-established differentiation potential (Takacs et al., 2023a).

Second, our culture system lacks additional components that characterize the native joint environment, such as synovial fluid-derived factors including hyaluronic acid, lubricin, and complex cytokine milieus. These factors modulate chondrocyte function, matrix homeostasis, and inflammation *in vivo*, and their absence in the culture medium may influence gene expression and phenotypic responses under mechanical unloading. Future studies incorporating co-culture systems or supplementation with joint-specific biofluids could enhance physiological relevance and model complexity.

Third, our study focuses primarily on transcriptional profiling, providing a comprehensive snapshot of gene expression dynamics. However, mRNA changes do not always translate directly to protein abundance or functional outcomes due to post-transcriptional regulation, protein turnover, and mechanochemical feedback. Complementary proteomic, biochemical, and biomechanical assessments are important future directions to validate and extend the mechanistic insights reported here.

Fourth, we did not determine the biological sex of the 4.5-day-old chick embryos used to derive limb bud progenitor cells, as sexing at this stage is incompatible with our dissection workflow. Consequently, our micromass cultures represent pooled male and female cells. This design precludes analysis of sex-specific responses but should avoid systematic bias toward a single sex and is consistent with common practice in embryonic limb bud micromass models (Takacs et al., 2023a; Underhill et al., 2014). Future work using sexed embryos or human sex-stratified donor material will be needed to directly address sex-dependent unloading responses.

Finally, while simulated microgravity via the RPM effectively models mechanical unloading, it differs from the complex mechanical, biochemical, and systemic factors present *in vivo* during disuse or OA progression. Therefore, caution is required in interpreting these results beyond the scope of basic mechanobiology. Nevertheless, by enabling precise control of unloading conditions and facilitating rapid detection of mechanosensitive molecular signatures, this platform presents a valuable system for dissecting fundamental principles of cartilage mechanotransduction and cellular plasticity.

### 4.2 Conclusion

In summary, our study demonstrates that simulated microgravity, applied through a RPM, constitutes a robust and rapid platform for probing fundamental mechanisms of cartilage biology, mechanotransduction, and lineage plasticity. We show that mechanical unloading induces both the suppression of chondrogenic identity genes and the activation of catabolic and ECM remodeling programs within a compressed timeframe of 72 h. Importantly, these transcriptional and phenotypic alterations are, to a degree, reversible, highlighting the inherent adaptability and plasticity of chondrogenic cells in response to dynamic mechanical cues.

Our findings align closely with recent systematic transcriptomic analyses of cartilage and chondroprogenitors under simulated microgravity (Wuest et al., 2015; Aleshcheva et al., 2013; Wehland et al., 2020), underscoring shared molecular signatures such as *SOX9* downregulation, BMP/TGF-β pathway dysregulation, and induction of MMPs. The RPM model thereby captures essential features of unloading-induced cartilage remodeling, providing a tunable and physiologically meaningful approach for dissecting temporal aspects of mechanobiology.

Given that mechanical forces play a central role in joint health and cartilage homeostasis (Juhasz et al., 2014; Bader et al., 2011), understanding the molecular cascades triggered by unloading advances our knowledge of cartilage degeneration and regeneration. The partial reversibility observed after transient microgravity exposure highlights potential windows of opportunity for therapeutic modulation of chondrocyte fate and ECM maintenance. Future integration of proteomic and biomechanical analyses will further elucidate the functional outcomes of these transcriptional programs.

Overall, simulated microgravity platforms represent valuable experimental models to accelerate investigations into cartilage mechanobiology and to explore novel mechanisms governing skeletal tissue plasticity under altered mechanical states.

## Data Availability

The datasets presented in this study can be found in online repositories. The names of the repository/repositories and accession number(s) can be found below: https://www.ncbi.nlm.nih.gov/bioproject/PRJNA1282631.
